# Neuroanatomy of Patients with Deficit Schizophrenia: An Exploratory Quantitative Meta-Analysis of Structural Neuroimaging Studies

**DOI:** 10.3390/ijerph17176227

**Published:** 2020-08-27

**Authors:** Tji Tjian Chee, Louis Chua, Hamilton Morrin, Mao Fong Lim, Johnson Fam, Roger Ho

**Affiliations:** 1 Department of Psychological Medicine, Yong Loo Lin School of Medicine, National University of Singapore, Singapore 119228, Singapore; johnson_fam@nuhs.edu.sg (J.F.); pcmrhcm@nus.edu.sg (R.H.); 2 Department of Psychological Medicine, National University Hospital, Singapore 119228, Singapore; 3 Yong Loo Lin School of Medicine, National University Singapore, Singapore 117597, Singapore; e0012747@u.nus.edu; 4 Department of Psychosis Studies, Institute of Psychiatry, Psychology & Neuroscience, King’s College London, London SE5 8AF, UK; morrinhamilton@gmail.com (H.M.); maofonglim@gmail.com (M.F.L.); 5 Guy’s & St Thomas’ NHS Foundation Trust, London SE11 4TX, UK; 6 East Suffolk and North Essex NHS Foundation Trust, Ipswich CO4 5JL, UK

**Keywords:** schizophrenia, deficit schizophrenia, negative symptoms, meta-analysis, systematic review, neuroanatomy, neuroimaging

## Abstract

Little is known regarding the neuroanatomical correlates of patients with deficit schizophrenia or persistent negative symptoms. In this meta-analysis, we aimed to determine whether patients with deficit schizophrenia have characteristic brain abnormalities. We searched PubMed, CINAHL and Ovid to identify studies that examined the various regions of interest amongst patients with deficit schizophrenia, patients with non-deficit schizophrenia and healthy controls. A total of 24 studies met our inclusion criteria. A random-effects model was used to calculate a combination of outcome measures, and heterogeneity was assessed by the *I*^2^ statistic and Cochran’s Q statistic. Our findings suggested that there was statistically significant reduction in grey matter volume (−0.433, 95% confidence interval (CI): −0.853 to −0.014, *p* = 0.043) and white matter volume (−0.319, 95% CI: −0.619 to −0.018, *p* = 0.038) in patients with deficit schizophrenia compared to healthy controls. There is also statistically significant reduction in total brain volume (−0.212, 95% CI: −0.384 to −0.041, *p* = 0.015) and white matter volume (−0.283, 95% CI: −0.546 to −0.021, *p* = 0.034) in patients with non-deficit schizophrenia compared to healthy controls. Between patients with deficit and non-deficit schizophrenia, there were no statistically significant differences in volumetric findings across the various regions of interest.

## 1. Introduction

The heterogeneity of schizophrenia has long captured the interest of researchers and clinicians alike. Considerable neuroanatomical, neurobiological and neuropsychological research has gone into discriminating between potential subtypes of schizophrenia characterized by the prevalence of symptom domains. In particular, negative symptoms, which may present as a deficit in goal-directed or pleasurable activity, speech and non-verbal expression [1], have been the source of some discussion, with Carpenter et al. [2] proposing the term deficit schizophrenia (DS) to describe the presence of primary and persistent negative symptoms [2].

DS has been suggested to differ from non-deficit schizophrenia (NDS) in its etiopathology, displaying association with impaired cognition, greater severity in course and poorer functional outcomes [3]. Reduced quality of life and impaired social and occupation functioning have also been reported in individuals with DS [4]. Previous population studies have suggested that the prevalence of DS amongst patients with schizophrenia is 15% in first episode psychosis, and 25–30% overall [5] and taxometric statistical analyses indicate that DS exists as a disease separately from NDS [6,7].

Diagnostic scales allowing one to reliably distinguish between DS and NDS are readily available, with the current gold standard being the Schedule for the Deficit Syndrome (SDS) [8]. For diagnosis of DS to take place, it is important to rule out secondary negative symptoms that may arise consequent to concurrent anxiety, depression, or extra-pyramidal side effects of medication. However, due to difficulty in distinguishing between primary and secondary symptoms, as well as the therapeutic relevance of each, the use of the more general descriptor of “persistent negative symptoms” (PNS) has been suggested by the National Institute of Mental Health-Measurement and Treatment Research to Improve Cognition in Schizophrenia (NIMH-MATRICS) to improve the homogeneity of clinical study populations [9,10,11]. Although PNS does not possess a bespoke diagnostic tool such as the SDS, a number of rating scales such as the Positive and Negative Symptom Scale (PANSS) [12], Scale for the Assessment of Negative Symptoms (SANS) [13] and Brief Negative Symptoms Scale (BNSS) [14] have been validated and are in use.

It has previously been suggested that negative symptoms of schizophrenia may be associated with certain structural changes [11,15]. However, research is ongoing to elucidate the neuropathological process of DS and PNS, with PNS having been described as an unmet therapeutic need meriting further study [9], particularly due to its resistance to current treatments.

Although several studies have sought to investigate the neuroanatomy of DS and PNS through imaging, they are limited in number and sample size when compared to NDS imaging studies [15]. Furthermore, direct comparison of these studies is hampered by differences in methodology, terminology, patient selection criteria and neuroimaging modality.

Despite the existence of reviews of DS and PNS neuroimaging research [11,15,16] as well as one meta-analysis of voxel-based morphometry (VBM) studies of the caudate nucleus in PNS [17], studies that were reviewed were inconsistent in terminology used for negative symptoms or did not all explicitly state the persistence of negative symptoms. Therefore, there is a need for a more comprehensive quantitative review and meta-analysis of global neuroanatomical changes in DS and PNS that distinguishes patients from healthy controls or NDS patients.

## 2. Materials and Methods

### 2.1. Search Strategy

A search strategy was conducted using the online databases OvidSP and CINAHL from July 2018 to August 2019. Keywords used included the terms ‘neuroimag’ OR ‘MRI’ OR ‘Magnetic resonance imag’ AND ‘deficit schizophreni’ OR ‘persistent negative symptom’ OR ‘non-affective psycho’. In OvidSP, the results were limited up to the year 2018, while the CINAHL results were limited up to July 2018.

### 2.2. Inclusion and Exclusion Criteria

Study inclusion criteria were as follows: studies that measured structural abnormalities using neuroimaging techniques included in an original paper in a peer-reviewed journal. Studies were case–control comparisons of neuroimaging studies investigating the neuroanatomy of patients with deficit schizophrenia. This included all magnetic resonance imaging studies with varied approaches of Region Of Interest (ROI), Voxel-Based Morphometry (VBM) and Diffusion Tensor Imaging (DTI). The wider inclusion criteria in this respect were necessary to increase the number of suitable publications. Deficit schizophrenia or persistent negative symptoms (PNS) were the main exposure/diagnosis. Brain structural correlate measurements with regional brain density and size as the outcome of interest. Comparison populations included patients with non-deficit schizophrenia, schizophrenic patients with little negative symptoms and/or normal controls. Participants in selected studies required a diagnosis of deficit schizophrenia, using at least one or more standardized assessment methods. Accepted diagnostic instruments included the following: The Schedule for Deficit Schizophrenia (SDS), the Persistent Negative Symptoms (PNS) classification, Proxy for the Deficit Syndrome (PDS), Positive and Negative Syndrome Scale (PANSS) and Scale for Assessment of Negative Syndrome (SANS). SDS is the gold standard, whilst PNS, PDS, PANSS and SANS were considered valid proxy assessments of deficit schizophrenia. Studies that were not written in English were excluded.

### 2.3. Data Extraction and Quality Assessment

Once a finalized list of relevant studies had been generated via the database search, initial screening of titles and abstracts was undertaken using a data collection and eligibility checklist sheet (see Appendix A Table A1) to decide which full papers should be included. Following verbal consensus on study inclusion, full-text articles were then collected and the data were extracted and compiled into a series of Excel spreadsheets for both systematic review and meta-analytic consideration. A database for demographic details and ROI examined by each individual study was created (see Appendix A Table A2) to enable gathering information on the number of studies that had examined a specific region of interest. Due to the variability in definitions of specific region of interest, specific quotes from the study outlining the region of interest examined were input into the database and compared. This process ensured that studies were accurately matched for specific region of interests, to prevent over-sampling error and bias. Regions of interests that were examined by more than one study were recruited into the review and statistical analysis. The mean and standard deviation values were then sought out and recorded into an Excel database (see Appendix A Table A3) for meta-analytic considerations. The following data were collected from the studies: the neuroimaging modality employed, i.e., magnetic resonance imaging—region of interest (ROI), voxel-based morphometry (VBM) or diffusion tensor imaging (DTI); the diagnostic instrument used to define deficit schizophrenia, i.e., the Schedule of Deficit Syndrome (SDS), the Persistent Negative Symptoms classification (PNS) or Scale for Assessment of Negative Symptoms (SANS)— although the Schedule of Deficit Syndrome is suggested as the gold standard in diagnosing deficit schizophrenia, studies that used certain proxy diagnostic instruments such as the Scale for Assessment of Negative Symptoms and Persistent Negative Symptoms to diagnose deficit schizophrenia were also accepted in order to increase the number of acceptable studies included in the meta-analysis; the number of deficit schizophrenia patients, non-deficit schizophrenia patients and healthy controls; the number and ratio of males to females in each study; the mean age of each group of DS, NDS and control patients in each study. For studies to be used in meta-analysis, we recorded all the mean and standard deviation values for the matched regions of interests.

### 2.4. Statistical Analyses

Statistical analyses were conducted while using the Comprehensive Meta-Analysis Version (CMA) 3.0 program. A random-effects model was adopted to calculate the continuous outcome measures from chosen studies and 95% confidence intervals (CIs) in view of the expected heterogeneity across the studies. Standard mean differences (SMD) were measured and referred to the Cohen’s effect size. Regions of interests with more than one study investigating this particular brain structure were included in statistical evaluation as long as suitable diagnostic instruments were implemented and continuous outcome measurements of means and standard deviation were recorded. The between-study heterogeneity was assessed by calculating the Cochran *Q* test statistic [18]. To assist with interpretation of between-study heterogeneity, the *I*^2^ statistic was also calculated. The *I*^2^ statistic was equivalent to the proportion of total variation across studies due to heterogeneity [19].

## 3. Results

**Figure 1:**
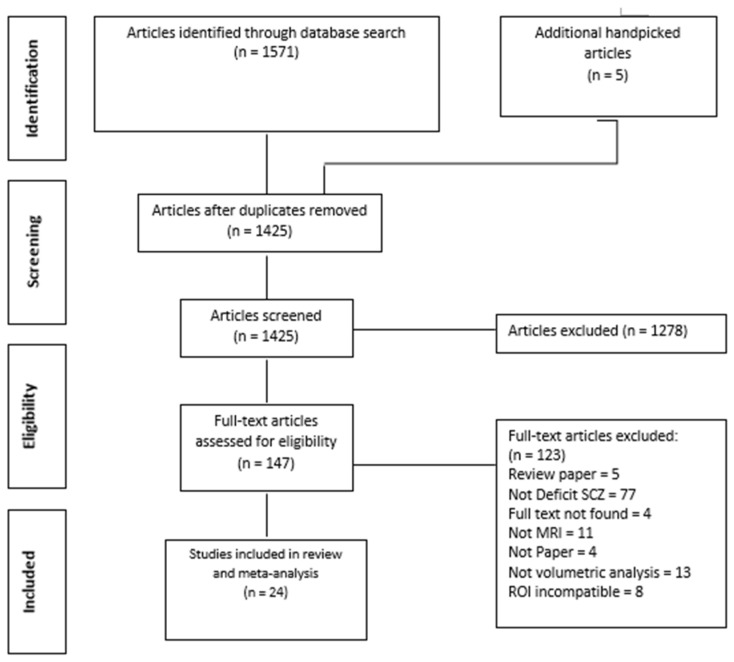
Process of systematic selection using the Preferred Reporting Items for Systematic Reviews and Meta-Analyses (PRISMA) flow chart.

Of the 1571 results that were obtained from the initial online electronic search and *x* results through other sources, a total of 24 studies were finally included in this review. The process of study selection is summarized with the Preferred Reporting Items for Systematic Reviews and Meta-Analyses (PRISMA) flow diagram, as depicted in Figure 1. All the final included studies were case–control in design and had utilized validated standardized instruments as methods to diagnose deficit schizophrenia. The various methods that were used in the final included studies were as follows: Schedule for Deficit Schizophrenia (SDS), Persistent Negative Symptoms (PNS) classification, Proxy for the Deficit Syndrome (PDS), Positive and Negative Syndrome Scale (PANSS) and the Scale for Assessment of Negative Syndrome (SANS). There were a total number of 2546 subjects included in this review consisting of 562 patients with deficit schizophrenia, 835 patients with non-deficit schizophrenia and 1149 healthy controls covered altogether. The demographic data and characteristic of each included article are presented in Appendix A Table A2.

### 3.1. Characteristics of Studies

A total of 24 studies were included in the systematic review process, eight of which were recruited for meta-analysis [20,21,22,23,24,25,26,27].

Of the identified region of interests, four brain structures have been examined by three or more independent studies with continuous quantitative data. A total of 12 meta-analytic comparisons took place between the deficit schizophrenia patient group, the non-deficit schizophrenia patient group and the healthy control group.

The demographic data of these 24 studies were entered into a database [20,21,22,23,24,25,26,27,28,29,30,31,32,33,34,35,36,37,38,39,40,41,42,43]. The variables of which are summarized in Table 1.

The mean age of patients with deficit schizophrenia ranged from 22.33 to 49.03 years. There was a mean of 23.4 deficit schizophrenia patients, 36.3 non-deficit schizophrenia patients and 47.9 healthy controls per study. This low deficit schizophrenia patient sample size is noted and may suggest the actual lower clinical sample prevalence. It may also indicate a sense of difficulty in diagnosing patients with deficit schizophrenia.

In the deficit schizophrenia patient group, the percentage of males was 77%. In all, 23 studies included both males and females, and there was only one paper that comprised of only male patients [29]. Roughly four-fifths of DS subjects were men, suggesting that males are much more commonly diagnosed with deficit schizophrenia than females are.

The primary diagnostic instruments used to define deficit schizophrenia in these papers were SDS [22,24,25,27,29,31,32,33,35,36,37,38,40,41], SANS [21,30,42,43], PNS [23], PANSS alongside The Diagnostic and Statistical Manual of Mental Disorders, Third Edition (DSM-III) and Fourth Edition (DSM-IV) criteria [26,34,39] and PDS [20,28]. In some studies, more than one instrument of diagnostic classification was used. SDS is the gold standard for diagnosing deficit schizophrenia with the high inter-rater reliability [8]. If SDS was not used in the study, other acceptable diagnostic instruments included the SANS, PNS, PANSS and PDS.

Overall, 13 studies employed the MRI ROI approach [20,22,24,26,27,28,29,30,36,40,41,42,43], six studies used VBM [21,23,25,31,33,39], four studies used diffusion tensor imaging (DTI) [34,35,37,38] and one study used both VBM and DTI [32].

### 3.2. Comparing Patients with Deficit Schizophrenia to Healthy Controls

Comparisons between the deficit schizophrenia patient group and the healthy controls across the four regions of interest were made and summarized in Table 2. The effect sizes of grey matter and white matter volumes in deficit schizophrenia compared against healthy controls (in bold) were statistically significantly smaller (effect size *p*-value less than 0.05).

**Figure 2:**
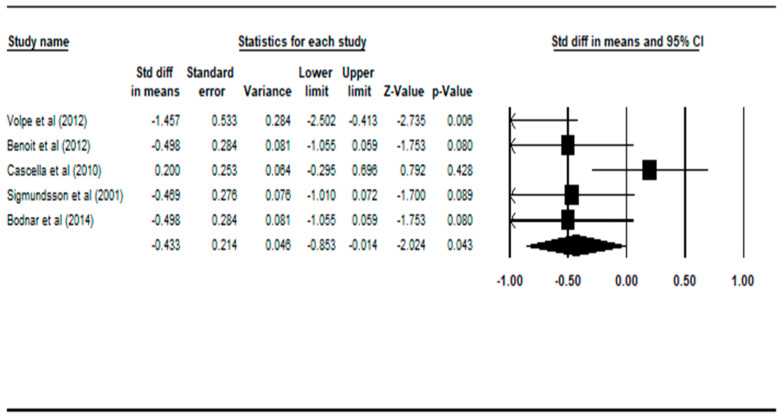
Forest plot: DS vs. HC, grey matter volume. Abbreviations: Std diff, standard difference; CI, confidence interval.

**Figure 3:**
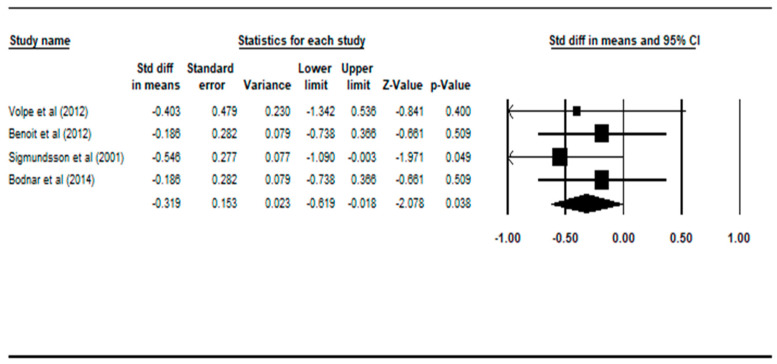
Forest plot: DS vs. HC, white matter volume.

Graphical representations of the statistically significant comparisons are plotted on the Forest plots in Figure 2 and Figure 3.

Compared with controls, patients with deficit schizophrenia had statistically significant smaller grey matter volumes, with a random effect size of −0.433 (95% CI: −0.853 to −0.014, *p* = 0.043), according to five studies [21,22,23,25,26].

In this particular comparison, there were four different diagnostic instruments used among the five studies. Two studies utilized the SDS [22,25], one study employed PNS [23], one study used PANSS [26] and one study used SANS [21] to define deficit schizophrenia in their patient group. This heterogeneous diagnostic process may affect inter-rater reliability, especially in the three studies that did not use SDS.

Compared with controls, patients with deficit schizophrenia had statistically significant smaller white matter volume, with a random effect size of −0.319 (95% CI: −0.619 to −0.018, *p* = 0.038), according to four studies [21,22,23,26].

Similar to the previous comparison, the diagnostic instruments used in all four studies were different to each other. One study utilized the SDS [22], one study employed PNS [23], one study used PANSS [26] and one study used SANS [21] to define deficit schizophrenia in their patient group. This heterogeneous diagnostic process may affect inter-rater reliability, especially in the three studies that did not use SDS.

### 3.3. Comparing Patients with Deficit Schizophrenia to Patients with Non-Deficit Schizophrenia

Comparisons between the deficit schizophrenia patient group and the non-deficit schizophrenia patient group across the four ROIs were made and summarized in Table 3. There appear to be no statistically significant differences in the effect sizes across the four regions of interest between patient groups of DS and NDS. As a result, we are not able to make any conclusions about the brain structural correlation changes between these two patient groups.

### 3.4. Comparing Patients with Non-Deficit Schizophrenia to Healthy Controls

**Figure 4:**
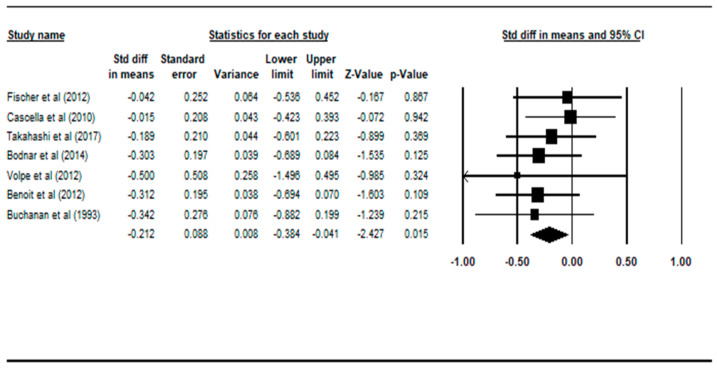
Forest plot: NDS vs. HC, total brain volume. Abbreviations: Std diff, standard difference; CI, confidence interval.

**Figure 5:**
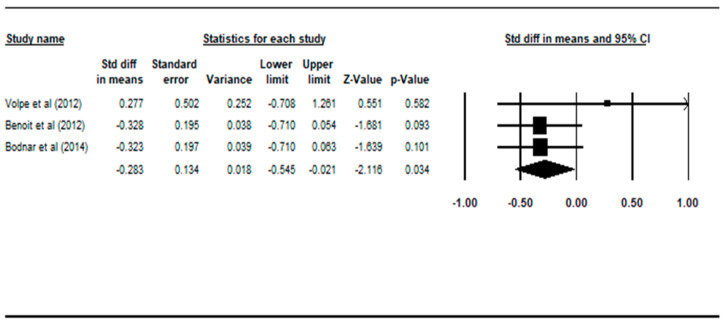
Forest plot: NDS vs. HC, white matter volume. Abbreviations: Std diff, standard difference; CI, confidence interval.

Comparisons between the non-deficit schizophrenia patient group and the healthy controls across the four regions of interest were made and summarized in Table 4. There were statistically significant findings in the total brain volume and white matter volume during comparison between patients with NDS versus healthy controls. Graphical representations of the statistically significant comparisons are plotted on the Forest plots in Figure 4 and Figure 5.

Compared with controls, patients with non-deficit schizophrenia had statistically smaller total brain volume, with an effect size of −0.212 (95% CI: −0.384 to −0.041, *p* = 0.015), according to seven studies [20,21,22,23,24,25,27].

Compared with controls, patients with non-deficit schizophrenia had statistically smaller white matter volume, with a random effect size of −0.283(95% CI: −0.546 to −0.021, *p* = 0.034), according to three studies [21,22,23].

## 4. Discussion

### 4.1. Deficit Schizophrenia versus Healthy Controls

In patients with deficit schizophrenia compared with healthy controls, we identified statistically significant reduced grey matter volume and reduced white matter volume.

### 4.2. Deficit Schizophrenia versus Non-Deficit Schizophrenia

In patients with deficit schizophrenia compared with those with non-deficit schizophrenia, there appeared to be no statistically significant differences in the effect sizes across the four brain regions investigated.

### 4.3. Non-Deficit Schizophrenia versus Healthy Control

In patients with non-deficit schizophrenia compared with healthy controls, we identified reduced total brain volume and decreased white matter volume.

### 4.4. Strengths and Limitations

The main strength of this study is that it is the first study to attempt to examine brain structural correlates in patients with deficit schizophrenia using a meta-analytic approach. With limited numbers of relevant studies so far, it is particularly important to ensure all related studies are considered. A methodical systematic approach to include all relevant studies was undertaken and achieved using a thorough and comprehensive search strategy.

Despite the strength of inclusion of relevant papers, the study has a number of significant limitations that should be taken into account prior to serious interpretation of the study findings. Regarding study design limitations, it became apparent during the data collection phase that the number of available and relevant neuroimaging studies that specifically addressed questions about the neuroanatomy of patients with deficit schizophrenia is relative scarce. For instance, the recent literature search revealed 24 studies relevant to deficit schizophrenia, whereas the systematic review study in 2001 by Shenton et al. [44] produced 180 studies. The sample in the Shenton et al. study [44] was mostly patients with chronic schizophrenia. The existing studies of patients with deficit schizophrenia tended to have a smaller patient sample. The existing average of 23.4 patients in this study is almost one third lower than the average of 33 patients per study reported in the systematic review by Shenton et al., 2001 [44]. In our meta-analysis, one out of the four region-of-interest comparisons that suggested statistical significance have three studies’ sample size. The other region-of-interest comparisons have between four and seven studies. The low number of studies, which translates to a small patient sample, per brain structure evaluated reduces the power of the analysis. An inadvertent limitation due to the small number of studies included in the meta-analysis would be that it is not possible to determine for publication bias, which may occur. The current lack of consensus among comparisons between studies studying the same region of interest is likely to reflect the generally low power of studies. In addition, for the four meta-analyses, the *p* values ranged from 0.015 to 0.043, and they would probably not be statistically significant if they were adjusted for multiple comparison.

Most brain volumetric studies included in this systematic review employed a region-of-interest approach (13 out of 24 studies). In this ROI approach, brain regions are outlined in an exacting manner, using pre-set operationalized procedures [45]. Due to this precise nature, it can create errors, because other region-of-interest outlines may not fulfil the specific description in another study. This error become magnified when a large volume of other similar but not exactly precise ROI were gathered together, in the case of a systematic review of many studies. In this present study, the specific description of brain regions of interest can differ between different authors and their papers. The lack of cohesion in describing the regions of interest measured between studies make direct comparisons of reported outcome measures difficult. This issue is compounded when authors use dissimilar labels to describe the same brain area [46,47]. Voxel-based morphometry studies are theoretically more favorable for meta-analytic processing. As group differences are described in standardized coordinates, meta-analytic techniques can be applied effectively. Although our search uncovered seven VBM studies, we were not able to utilize them for quantitative analysis.

There were also clinical limitations faced in this study. Deficit schizophrenia is described as “a set of primary, enduring negative symptoms of schizophrenia”. However, there often exists a complicated heterogeneity between primary and secondary symptoms of negative schizophrenia. The mean age of the patient group with deficit schizophrenia in this study was 33.4 years old. However, the age group ranged from 24 to 40 years old. This wide age range may introduce confounding factors that may affect the accuracy of diagnosis of deficit schizophrenia. For example, an older patient with deficit schizophrenia is more likely to develop negative symptoms secondary to the use of antipsychotics or become affected by psychosocial circumstances. The diagnostic instrument used in defining deficit schizophrenia has not been singularly standardized. Experts differ in their opinions regarding these scales. Some recommend the Schedule of Deficit Schizophrenia (SDS) as the current gold standard for diagnosing deficit schizophrenia. However, only 14 of the 24 studies (58.3%) in our review used the Schedule of Deficit Schizophrenia as a diagnostic tool. In the comparisons involving patients with deficit schizophrenia and healthy controls, there was heterogeneity in the diagnostic instrument used to diagnose patients with deficit schizophrenia, thereby affecting inter-rater reliability, especially in studies that did not use SDS. For patients with schizophrenia, both the deficit and the non-deficit form, one typical scenario is that they will be rapidly started on some form of neuroleptic medication soon after diagnosis. Different types and dosages of medications will be prescribed, presenting with significant treatment heterogeneity. Questions should be asked about the timing as well as the cause of brain volume changes, particularly in studies that show statistically significant findings. Volumetric changes occurring for reasons other than those related to the pathophysiology of deficit schizophrenia are likely to cause Type 1 errors or false-positive outcomes. Older patients are more likely to develop volumetric changes due to secondary causes of negative symptoms (for example, antipsychotic medications). In younger patients with true deficit schizophrenia, the rate of volumetric loss may be insufficient for detection by either the MRI or during analytical cutoffs in this study.

Lastly, we also encountered imaging limitations in this study. Since this study involved only the MRI modality, it is important to discuss potential pitfalls and difficulties with the use of MRI volumetric measuring methods [48]. Different MRI software or machinery operation can lead to volumetric changes of up to 5% [49]. Calculation errors, both manual and computerized, occurring during neuroimaging processing can average approximately 1.5%, and even though this inaccuracy can be adjusted for, neglecting its adjustment can lead to systematic error, and ultimately reduce the level of agreement amongst the various studies. In MRI studies employing voxel-based morphometry, imprecision due to the misclassification of voxels occurring during brain segmentation is one of the more common causes of imaging error [50]. Grey matter proximity with cerebrospinal fluid can lead to a poorly defined edge and cause volume estimation errors [50]. Poor positioning of the head or of the imaging slab can cause inaccuracy in brain volume measurements [48]. The only way to resolve this issue fully is to aim for full-brain coverage during an examination. One of the last but important imaging limitations likely to be encountered in MRI volumetric measurements is random mistakes or miscalculations, that are often out of the control of the technician. These are non-systemic errors [51] and may be significant.

## 5. Conclusions

The most statistically significant volumetric findings in our study of patients suggest that compared with healthy normal controls, patients with deficit schizophrenia have reduced grey and white matter volumes (Table 2), while patients with non-deficit schizophrenia have reduced total brain volume and white matter volume (Table 4). Between patients with deficit and non-deficit schizophrenia, there were no statistically significant differences in volumetric findings across the four brain regions (Table 3).

However, these observed measure outcomes of brain structural changes should not be conclusive due to significant limitations on the study design, particularly in the areas of small sample sizes and limited studies examining the neuroanatomy of deficit schizophrenia. Inconsistencies of imaging technique and the likelihood of a less homogeneous patient sample also contribute to this caution.

This review is an exploratory first-investigation into this topic. It re-affirms the need for further research into the neuroanatomy of deficit schizophrenia. Perhaps with the relatively low level of involvement so far, this is an area of promise.

However, the traditional complexities and barriers that turn away prospective researchers needed to be addressed first: The first lies in diagnosing deficit schizophrenia in the patient. A gold standard diagnostic instrument, currently the Schedule of Deficient Syndrome (SDS), should be used whenever possible because it has the highest level of inter-rater reliability, which ultimately aids research and subsequent reviews. The process of using the SDS is tedious, but the rewards would be worthwhile. The second lies in meticulous study design to improve the power of the study and minimize confounders. Recruitment of larger independent samples and careful sampling criteria to focus on a more homogeneous group of patients with primary negative symptoms by controlling risk factors for secondary negative symptoms such as old age, long duration of mental illness, antipsychotic medications, etc. should also be employed.

## Figures and Tables

**Table 1:** Summary of studies included in systematic review and meta-analysis.

Study (Year)	Sample Size			Patient Age Mean (Years)	Male (DS) %	Diagnostic Criteria	Imaging Modality
	DS Group *n*	NDS Group *n*	Control Group *n*				
Takayanagi et al. (2018)	37	36	50	25.8	50	PDS	MRI (ROI)
Xie et al. (2017)	33	41	41	49.03	100	SDS	MRI (ROI)
Makowski et al. (2017)	21 (early PNS)	44	44	23.2	71.4	SANS	MRI (ROI and MAGeT)
Takahashi et al. (2017)	38	37	59	27.1	57.9	PDS	MRI (ROI)
De Rossi et al. (2016)	22	22	22	39.2	77.3	SDS	MRI (VBM)
Lei et al. (2015)	33	42	41	22.33	66.6	SDS	MRI (VBM and DTI)
Lei et al. (2015)	44	44	44	23.16	59.1	SDS	MRI (VBM)
Bodnar et al. (2014)	16 (PNS)	46	60	24.2	81.3	SANS	MRI (VBM)
Voineskos et al. (2013)	18	59	79	49	77.7	PANSS	MRI (DTI)
Takayanagi et al. (2013)	18	30	82	35.9	66.6	SDS	MRI (DTI)
Volpe et al. (2012)	10	8	8	35.8	90.0	SDS	MRI (ROI)
Özdemir et al. (2012)	11	18	17	32.36	63.6	SDS	MRI (ROI)
Benoit et al. (2012)	16	48	60	24.2	81.3	PNS	MRI (VBM)
Kitis et al. (2012)	11	18	17	32.36	63.6	SDS	MRI (DTI)
Fischer et al. (2012)	20	36	28	40.1	85.0	SDS	MRI (ROI)
Cascella et al. (2010)	19	31	90	35.1	84.2	SDS	MRI (VBM)
Rowland et al. (2009)	10	10	11	43	84.2	SDS	MRI (DTI)
Koutsouleris et al. (2008)	59	106	177	32.8	84.7	PANSS	MRI (VBM)
Galderisi et al. (2008)	34	32	31	35.8	73.5	SDS	MRI (ROI)
Quarantelli et al. (2002)	14	14	25	NR	92.9	SDS	MRI (ROI)
Sigmundsson et al. (2001)	27	0	27	34.9	96.3	PANSS	MRI (ROI)
Sanfilippo et al. (2000)	13	40	29	NR	NR	SANS	MRI (ROI)
Turetsky et al. (1995)	21	49	77	NR	85.7	SANS SCOS	MRI (ROI)
Buchanan et al. (1993)	17	24	30	35.5	NR	SDS	MRI (ROI)
Total	562	835	1149				
Mean	23.4	36.3	47.9	33.4	77.0		

Abbreviations: DS = deficit schizophrenia; NDS = non-deficit schizophrenia; NR = not reported; SDS = Schedule for Deficit Schizophrenia; PANSS = Positive and Negative Syndrome Scale; PNS = Persistent Negative Symptoms (PNS) classification; SCOS = Strauss–Carpenter Outcome Scale; PDS = Proxy for the Deficit Syndrome; MRI = stereotaxy-based regional brain volumetry applied to segmented MRI.

**Table 2:** Meta-analysis of continuous data comparing patients with DS vs. healthy controls (HC).

			DS Patients vs. Controls		Heterogeneity		
Region	No of Studies	No. of DS/HC	Effect Size (95% CI)	Effect Size *p* Value	Q	I^2^ (%)	*p* Value
TBV	8	163/362	−0.161 (−0.362 to 0.040)	0.117	7.56	7.4	0.373
GM	5	88/215	−0.433 (−0.853 to −0.014)	0.043	9.78	59.1	0.044
WM	4	69/155	−0.319 (−0.619 to −0.018)	0.038	1.15	0.0	0.765
CSF	5	89/183	0.107 (−0.158 to 0.373)	0.428	1.81	0.0	0.771

Abbreviations: DS, deficit schizophrenia; HC, healthy control; CI, confidence interval; TBV, total brain volume; GM, grey matter; WM, white matter; CSF, cerebrospinal fluid.

**Table 3:** Meta-analysis of continuous data comparing patients with DS vs. NDS.

			DS Patients vs. NDS Patients		Heterogeneity		
Region	No of Studies	No. of DS/NDS	Effect Size (95% CI)	Effect Size *p* Value	Q	I^2^ (%)	*p* Value
TBV	7	136/230	0.066 (−0.152 to 0.283)	0.554	2.94	0.0	0.816
GM	4	61/133	−0.061 (−0.409 to 0.287)	0.732	3.67	18.3	0.299
WM	3	42/102	−0.046 (−0.533 to 0.440)	0.852	3.19	37.4	0.203
CSF	4	62/138	0.121 (−0.185 to 0.426)	0.439	0.021	0.0	0.999

Abbreviations: DS, deficit schizophrenia; NDS, non deficit schizophrenia; CI, confidence interval, TBV, total brain volume; GM, gray matter; WM, white matter; CSF, cerebrospinal fluid.

**Table 4:** Meta-analysis of continuous data comparing patients with non-deficit schizophrenia vs. healthy controls (HC).

			NDS Patients vs. Controls		Heterogeneity		
Region	No of Studies	No. of NDS/HC	Effect Size (95% CI)	Effect Size *p* Value	Q	I^2^ (%)	*p* Value
TBV	7	230/332	−0.212 (−0.384 to 0.041)	0.015	2.38	0.0	0.882
GM	4	133/218	−0.272 (−0.566 to 0.022)	0.070	4.76	36.9	0.191
WM	3	102/128	−0.283 (−0.545 to −0.021)	0.034	1.34	0.0	0.513
CSF	4	138/156	−0.113 (−0.344 to 0.118)	0.337	0.97	0.0	0.808

Abbreviations: NDS, non deficit schizophrenia; HC, healthy control; CI, confidence interval, TBV, total brain volume; GM, gray matter; WM, white matter; CSF, cerebrospinal fluid.

## Appendix A

**Table A1:** Data extraction sheet.

First Author	Year		PubMed ID	
Included in Review? (circle response)	Yes		No	
Reason for exclusion:				
MRI (circle response)	ROI	VBM	DTI	Others:
Diagnostic instrument:				
Deficit Schizophrenia Patient Group	Number M:F Mean age		ROIs included in paper:	
Non-Deficit Schizophrenia Patient Group	Number M:F Mean age			
Healthy Controls	Number M:F Mean age			

MRI = stereotaxy-based regional brain volumetry applied to segmented MRI. ROI = Region Of Interest. VBM = voxel-based morphometry. DTI = Diffusion Tensor Imaging.

**Table A2:** Data extraction sheet—demographics and ROI.

	Study (Year)	Struct Imaging	Relevant Diagnostic Instrument	DS or PNS		Non-DS or Non-PNS		Controls		Regions of Interest
		MRI		N (M:F)	Mean/Median Age	N (M:F)	Mean/Median Age	N (M:F)	Mean/Median Age	
1	Volpe (2012)	ROI (grey matter volumes)	Schedule for Deficit Syndrome (SDS)	10 (9:1)	35.8	8 (7:1)	34.2	8 (7:1)	33	Hippocampus
										Dorsolateral Prefrontal Cortex (DLPFC)
										GM
										WM
										ICV
										CSF
2	Özdemir (2012)	ROI	SDS (Turkish version)	11 (7:4)	32.4	18 (9:9)	40.8	17 (9:8)	33.82	Left DLPFC,
										Right Superior Temporal Gyrus (STG),
										Left STG,
										Right anterior Prefrontal Cortex (PFC),
										Left DLPFC,
										Culmen,
										Right Frontal Eye Field (FEF),
										Right temporopolar cortex,
										Left Middle Temporal Gyrus (MTG),
										Right inferior PFC,
										Left posterior cingulate,
										Left anterior PFC,
										Left parahippocampal gyrus,
										Left angular gyrus
										GM
										WM
										CSF
										TBV
3	Benoit (2012)	VBM	Persistent Negative Symptoms (PNS) classification	16 (13:3)	24.2	48 (33:15)	23.6	60 (40:20)	24.8	Frontal cortex,
										Temporal lobe,
										Cingulate cortex,
										Caudate,
										Putamen,
										Globus pallidus,
										Amygdala–Hippocampus,
										Hippocampus,
										Ventricles
										GM
										WM
										CSF
4	Kitis (2012)	DTI	SDS	11 (7:4)	32.36	18 (9:9)	40.77	17 (9:8)	33.82	Fractional anisotropy in uncinate fasciculus, left and right.
5	Fischer (2012)	ROI	SDS, SANS, DSM-IV	20 (17:3)	40.1	36 (31:5)	38.4	28 (23:5)	36	DPFLC Circuit Regions
										- Middle frontal gyrus grey matter
										- supramarginal gyrus grey matter
										- thalamus
										- caudate
										Non-DPFLC Circuit Regions
										- superior frontal gyrus grey matter
										- inferior frontal gyrus grey matter
										- orbital frontal gyrus grey matter
										- superior temporal gyrus grey matter
										- amygdala–hippocampal complex
										- middle temporal gyrus grey matter
										Total Cranial Vol.
										Total Brain Vol. (TBV)
										Total Ventricular Vol.
										Total CSF Vol.
6	Cascella (2010)	VBM	SDS, DSM-IV, SANS	19 (16:3)	35.1	31 (21:10)	44.4	90 (43:47)	46.3	VBM analyses of grey matter volumes
										Frontal
										Temporal
										Sub-lobar
										Limbic
										Occipital
										Cerebellum
										GM
										TBV
7	Rowland (2009)	DTI (white matter alterations)	SDS, DSM-IV, SANS	10 (8:2)	43	10 (8:2)	40	11 (8:3)	37	Middle Frontal and Inferior Parietal White Matter Volume and Fractional Anisotropy (FA)
8	Galderisi (2008)	ROI	SDS, DSM-IV	34 (25:9)	35.8	32 (26:6)	34.2	31 (21:10)	34.4	Right, Left lateral ventricle,
										Right, Left DLPFC,
										Right, Left Hippocampus,
										Right, Left Cingulate cortex,
										Right, Left Temporal Lobe,
										Right, Left Putamen,
										Right, Left Pallidum,
										Right, Left Caudate
9	Quarantelli (2002)	Stereotaxy-based regional brain volumetry applied to segmented MRI.	SDS, SANS	14 (13:1)	20−51	14 (13:1)	19−54	25 (19:6)	18−50	Cerebellum
										Frontal
										Occipital
										Parietal
										Temporal
										Lateral ventricles
10	Sigmundsson (2001)	ROI	DSM-IV, PANSS, SDS.	27 (26:1)	34.9	-	-	27 (25:2)	32.2	Whole brain
										GM
										WM
										CSF
										Grey matter “deficit” region:
										- Perisylvian region
										- Medial frontal lobe/anterior cingulated
										- Parahippocampal gyrus
11	Sanfilipo (2000)	MRI	DSM-III, SANS	*n* = 13 (High negative symptom group)				29	35.8	24 ROIs for NDS vs. HC only.
										Superior Medial Prefrontal Grey
										Superior Central Prefrontal Grey
										Superior Lateral Prefrontal Grey
										Inferior Medial Prefrontal Grey
										Inferior Central Prefrontal Grey
										Inferior Lateral Prefrontal Grey
										Hemispheric Prefrontal Grey
										Total Prefrontal Grey
										Superior Medial Prefrontal White
										Superior Central Prefrontal White
										Superior Lateral Prefrontal White
										Inferior Medial Prefrontal White
										Inferior Central Prefrontal White
										Inferior Lateral Prefrontal White
										Hemispheric Prefrontal White
										Total Prefrontal White
										Hippocampus
										Parahippocampus
										Superior Temporal Gyrus
										Hemispheric Whole Temporal GM
										Total Whole Temporal GM
										Hemispheric Whole Temporal WM
										Total Whole Temporal WM
12	Turetsky (1995)	MRI	SANS, Strauss–Carpenter Outcome Scale	21 (18:3)	22.8	49 (26:23)	23.2	77 (48:29)	28	Regional Volumetric Measurements:
										Left Temporal
										Right Temporal
										Left Frontal
										Right Frontal
13	Takayanagi (2018)	ROI	PDS	37 (21:16)	27.2	36 (12:24)	26.6	50 (25:25)	25.8	Local gyrification index of:
										Right, Left dorsal medial prefrontal cortex,
										Right, Left ventromedial prefrontal cortex,
										Right, Left anterior cingulate gyrus,
										Right, Left superior frontal cortex,
										Right, Left medial orbitofrontal gyrus,
										Right, Left lateral orbitofrontal gyrus,
										Right, Left rostral anterior cingulate gyrus,
										Left postcentral gyrus,
										Left lingual gyrus,
										Right posterior cingulate gyrus,
										Right inferior parietal lobule
										Right lateral occipital cortex
14	Xie (2017)	ROI	SDS	33 (33:0)	49	41 (41:0)	45.1	41 (41:0)	45.8	Left superior temporal gyrus,
										Right superior temporal gyrus,
										Left middle temporal gyrus,
										Right middle temporal gyrus,
										Left inferior frontal gyrus triangular part,
										Right inferior frontal gyrus triangular part,
										Left Heschl gyrus,
										Left supramarginal gyrus,
										Left angular gyrus
										Left superior temporal gyrus temporal pole,
										Right inferior frontal gyrus orbital part,
										Left Insula
15	Makowski (2017)	ROI	SANS	21 (15:6)	23.2	44 (31:13)	24.6	44 (25:19)	23.8	Left Amygdala,
										Right Amygdala,
										Left Hippocampus,
										Right Hippocampus,
16	Takahashi (2017)	ROI	PDS	38 (22:16)	27.1	37 (12:25)	27.1	59 (28:31)	26.1	Left OFC (Orbitofrontal cortex),
										Right OFC,
										Left IOS (Intermediate orbital sulcus),
										Right IOS,
										Left POS (Posterior orbital sulcus),
										Right POS,
										CSP (Cavum septum pellucidi) volume,
										Olfactory sulcus depth,
										Intracranial volume
										Left superior temporal gyrus,
										Right superior temporal gyrus,
										Left middle temporal gyrus,
										Right middle temporal gyrus,
										Left inferior frontal gyrus triangular part,
										Right inferior frontal gyrus triangular part,
										Left Heschl gyrus,
										Left supramarginal gyrus,
										Left angular gyrus
										Left superior temporal gyrus temporal pole,
										Right inferior frontal gyrus orbital part,
										Left Insula
17	De Rossi (2016)	ROI	SDS	22 (17:5)	39.2	22 (17:5)	38.3	22 (17:5)	38.3	Left Accumbens,
										Left Thalamus,
										Left Caudate,
										Left Putamen,
										Left Pallidum,
										Left Amygdala,
										Right Accumbens,
										Right Thalamus,
										Right Caudate,
										Right Putamen,
										Right Pallidum,
										Right Amygdala,
										Intracranial volume
										Left superior temporal gyrus,
										Right superior temporal gyrus,
										Left middle temporal gyrus,
										Right middle temporal gyrus,
										Left inferior frontal gyrus triangular part,
										Right inferior frontal gyrus triangular part,
										Left Heschl gyrus,
										Left supramarginal gyrus,
										Left angular gyrus
										Left superior temporal gyrus temporal pole,
										Right inferior frontal gyrus orbital part,
										Left Insula
18	Lei (2015)	VBM/DTI	SDS	33 (21:11)	22.3	42 (25:17)	23.4	41 (24:17)	3.5	White matter:
										Precentral gyrus,
										Cerebellum posterior lobe,
										Extra-nuclear,
										Insula,
										total white matter volume,
										whole brain volume
										Left superior temporal gyrus,
										Right superior temporal gyrus,
										Left middle temporal gyrus,
										Right middle temporal gyrus,
										Left inferior frontal gyrus triangular part,
										Right inferior frontal gyrus triangular part,
										Left Heschl gyrus,
										Left supramarginal gyrus,
										Left angular gyrus
										Left superior temporal gyrus temporal pole,
										Right inferior frontal gyrus orbital part,
										Left Insula
19	Lei (2015)	ROI	SDS	44 (26:18)	22.9	44 (26:18)	23.2	44 (26:18)	22.6	Grey matter volume:
										Cerebellar culmen,
										Insula,
										total grey matter volume,
										Whole brain volume
										Left superior temporal gyrus,
										Right superior temporal gyrus,
										Left middle temporal gyrus,
										Right middle temporal gyrus,
										Left inferior frontal gyrus triangular part,
										Right inferior frontal gyrus triangular part,
										Left Heschl gyrus,
										Left supramarginal gyrus,
										Left angular gyrus
										Left superior temporal gyrus temporal pole,
										Right inferior frontal gyrus orbital part,
										Left Insula
20	Bodnar (2014)	ROI	SANS	16 (13:3)	24.2	46 (32:14)	23.7	60 (40:20)	24.8	Grey matter,
										White matter,
										CSF,
										Total intracranial
										Right Medial frontal gyrus,
										Right Orbital frontal gyrus,
										Right Anterior cingulate,
										Right Parahippocampal gyrus,
										Right Inferior temporal gyrus,
										Right Anterior/middle cingulate,
										Right, Left Middle temporal gyrus,
										Right, Left Superior temporal gyrus,
										Right Posterior cingulate,
										R,L Fusiform gyrus,
										Right Middle occipital gyrus,
										Left Inferior frontal gyrus,
										Left Middle frontal gyrus,
										Left Subgenual cingulate,
										Left Cuneus,
										Left Lingual gyrus
21	Voineskos (2013)	DTI	PANSS	18 (14:4)	49	59 (38:21)	43	79 (48:31)	43	White matter tract
										Left, Right inferior longitudinal fasciculus,
										Left, Right arcuate fasciculus,
										Left, Right uncinate fasciculus,
										Left, Right inferior occipitofrontal fasciculus,
										Left, Right cingulum bundle,
										Genu corpus callosum,
										Splenium corpus callosum
										Cortical region:
										Orbitofrontal cortex,
										middle temporal gyrus,
										superior temporal gyrus, temporal pole, DLPFC,
										Parietal operculum,
										parahippocampal gyrus,
										Insula
22	Takayanagi (2013)	ROI	SDS	18 (15:3)	35.9	30 (20:10)	44.3	82 (40:42)	43.7	Left, Right anterior cingulate grey matter volume,
										Left, Right anterior cingulate cortical thickness,
										Left, Right anterior cingulate surface area
23	Koutsouleris (2008)	VBM	PANSS	59 (50:9)	32.8	NR	NR	177 (123:54)	31.5	Perisylvian and Intrasylvian,
										Temporal,
										Frontal,
										Limbic,
										Thalamus and Basal ganglia,
										GM,
										WM,
										CSF,
										Total intracranial volume,
24	Buchanan (1993)	ROI	SDS	17 (12:5)	35.5	24 (14:10)	35.6	30 (20:10)	34	ROIs included in paper:
										Left, Right prefrontal total volume,
										Left, Right prefrontal grey matter volume,
										Left, Right prefrontal white matter volume,
										Left, Right caudate total volume,
										Left, Right amygdala/hippocampus total volume,
										Total cranial volume

**Table A3:** Mean and SD values for ROIs with more than three studies.

	Brain Structure	Study	Brain Structure Description (Quotation from Paper)	No. of DS Pts	No. of NDS Pts	No. of Controls	Units Used	Total Mean Vol. (DS)	Total Vol. SD (DS)	Total Mean Vol. (NDS)	Total Vol. SD (NDS)	Total Mean Vol. (cn)	Total Vol. SD (cn)
1. TBV	TBV	Fischer (2012)	Did not specify	20	36	28	mL	1299.2	43.9	1331.1	33.6	1339.2	34
	TBV	Cascella (2010)	Did not specify	19	31	90	mL	1209	149	1155	118	1157	137
	“Whole Brain”	Sigmundsson (2001)	Did not specify	27	0	27	mL	1298	122			1358	136
	ICV	Takahashi (2017)	Did not specify	38	37	59	mL	1472.9	153.2	1459	150.2	1487.2	148.7
	TIV	Bodnar (2014)	Did not specify	16	46	60	mL	1430	127	1437	121	1479	151
	ICV	Volpe (2012)	Did not specify	10	8	8	cc	1302.6	126.59	1316.34	125.65	1374.75	107.1
	TIV	Benoit (2012)	Did not specify	16	48	60	mL	1430	127	1436	119	1479	151
	TCV	Buchanan (1993)	Did not specify	17	24	30	cc	1229	153	1188	153	1234	118
2. Total Grey Vol.	GM	Volpe (2012)	Did not specify	10	8	8	mL	655.15	52.78	641.48	74.18	727.76	45.73
	Grey matter (mL)	Benoit (2012)	Did not specify	16	48	60	mL	624	56	642	59	658	71
	Grey matter (mL)	Cascella (2010)	Did not specify	19	31	90	mL	706	89	679	76	688	90
	Grey matter (mL)	Sigmundsson (2001)	Did not specify	27	0	27	mL	509	55			538	68
	Grey matter (mL)	Bodnar (2014)	Did not specify	16	46	60	mL	624	56	643	60	658	71
3. Total White Matter	WM.	Volpe (2012)	Did not specify	10	8	8	mL	489.31	45.31	525.97	44.83	510.9	62.63
	WM.	Benoit (2012)	Did not specify	16	48	60	mL	605	65	596	62	618	71
	WM.	Sigmundsson (2001)	Did not specify	27	0	27	mL	586	67			624	72
	WM.	Bodnar (2014)	Did not specify	16	46	60	mL	605	65	596	64	618	71
4. CSF	CSF	Volpe (2012)	Did not specify	10	8	8	mL	158.14	73.5	148.89	47.85	136.09	43.89
	CSF	Benoit (2012)	Did not specify	16	48	60	mL	201	27	198	27	203	35
	Total CSF vol	Fischer (2012)	Did not specify	20	36	28	mL	114.6	10.4	110.1	7.9	110.6	8
	CSF	Sigmundsson (2001)	Did not specify	27	0	27	mL	161	34			150	27
	CSF	Bodnar (2014)	Did not specify	16	46	60	mL	201	27	197	27	203	35
